# Very low‐calorie ketogenic diet and liraglutide as a synergistic strategy for the treatment of obesity: A short‐term, non‐randomised, observational, real‐world clinical evaluation

**DOI:** 10.1111/dom.16658

**Published:** 2025-07-30

**Authors:** Elisabetta Camajani, Davide Masi, Maria Letizia Spizzichini, Camilla Cori, Rebecca Rossetti, Maria Elena Spoltore, Dario Tuccinardi, Carla Lubrano, Lucio Gnessi, Andrea M. Isidori, Daniele Gianfrilli, Costanzo Moretti, Massimiliano Caprio, Mikiko Watanabe

**Affiliations:** ^1^ Laboratory of Cardiovascular Endocrinology IRCCS San Raffaele Rome Italy; ^2^ Department for the Promotion of Human Sciences and Quality of Life San Raffaele Open University Rome Italy; ^3^ Department of Experimental Medicine, Section of Medical Pathophysiology, Food Science and Endocrinology Sapienza University of Rome Rome Italy; ^4^ Research Unit of Endocrinology and Diabetes Department of Medicine and Surgery, Università Campus Bio‐Medico di Roma Rome Italy; ^5^ Fondazione Policlinico Universitario Campus Bio‐Medico Rome Italy; ^6^ Department of Systems' Medicine University of Rome Tor Vergata Rome Italy

**Keywords:** adverse events, anti‐obesity medication, GLP‐1 analogue, GLP‐1 receptor agonist, hypoglycaemia, side effects, very low‐carbohydrate diet, very low‐energy ketogenic therapy, VLCKD, VLEKT, weight loss

## 1 INTRODUCTION

Obesity is caused by multiple factors leading to excessive fat accumulation. The Very Low‐Calorie Ketogenic Diet (VLCKD), now renamed Very Low‐Energy Ketogenic Therapy,^1^ leads to weight loss through enhanced satiety via sustained ghrelin suppression,^2^ while reducing muscle loss.^3^, ^4^ As long‐term maintenance through diet is challenging, pharmacotherapy is often indicated.^5^, ^6^

Liraglutide, a glucagon‐like peptide‐1 receptor agonist (GLP‐1 RA), reduces appetite via ghrelin‐independent mechanisms. Interestingly, circulating ghrelin levels may increase during treatment, potentially counteracting its anorectic effects.^7^, ^8^ Moreover, GLP‐1 RAs may cause gastrointestinal adverse events and might lead to inadequate protein intake if appetite is excessively reduced.^9^, ^10^

Combining a protein‐rich VLCKD with liraglutide could, therefore, provide synergistic appetite suppression via both ghrelin‐dependent and ghrelin‐independent pathways, potentially resulting in greater weight loss. However, this could come at the cost of increased hypoglycaemia risk due to carbohydrate restriction.^11^ Current guidelines acknowledge ketogenic diets even for type 2 diabetes,^12^ but recommendations on combining GLP‐1 RAs with VLCKD are lacking, and, to the best of our knowledge, no published study has evaluated this specific combination in a real‐world clinical setting. Therefore, we aimed to compare weight loss, metabolic effects and safety between VLCKD alone and VLCKD combined with liraglutide.

## 2 METHODS

This prospective study compared a VLCKD alone versus VLCKD combined with liraglutide (VLCKD+Lira) over 4 months. Adult participants (18–65 years, BMI >30 kg/m^2^, stable weight in the last 3 months) freely choose their treatment, with stratification by age, gender and BMI. Key exclusions included severe organ failure, insulin‐dependent diabetes, pregnancy, psychiatric disorders affecting adherence or contraindications to GLP‐1 RA therapy. The study protocol was approved by Sapienza University Ethics Committee (ref. 5475), and participants provided written informed consent.

All participants, both in the VLCKD group and the combined VLCKD + liraglutide group, followed a very low‐calorie ketogenic diet with meal replacements for at least 45 days. The nutritional intervention provided approximately 800 kcal/day and consisted of 4–5 meal replacements daily, alongside one portion of low‐glycaemic index vegetables at lunch and dinner. The number of meal replacements was adjusted according to sex—four per day for women and five for men—to ensure adequate protein intake. The macronutrient composition included <50 g of carbohydrates daily, 1.2–1.5 g of protein per kg of ideal body weight, with fat making up the remaining caloric intake.^1^ Meal replacements were then gradually replaced by protein‐based dishes composed primarily of egg, lean meat and fish, with extra virgin olive oil as the primary fat source. Caloric intake was progressively increased to approximately 1200 kcal/day. All participants were advised to drink at least 2 litres of water daily and received multivitamin and mineral supplementation according to current EFSA recommendations.^13^ No dietary carbohydrates were introduced until the end of the 4‐month observation period. Participants in the VLCKD + liraglutide group were prescribed liraglutide in addition to the dietary intervention. All participants were naïve to GLP‐1 receptor agonists at baseline. The initial dose was 0.6 mg daily, which was gradually uptitrated to a maximum of 1.8 mg based on individual patient tolerance. Dose adjustments were made under close medical supervision to ensure safety and optimise treatment efficacy.

Clinical (weight, BMI waist circumference) and laboratory (lipids, glucose, insulin, electrolytes, renal and liver function, uric acid and HOMA‐IR) assessments were performed in a fasting state at baseline and after 4 months. Safety and adherence were monitored via dietitian contact through scheduled in‐person visits, with additional remote follow‐ups as needed. Adherence was assessed via confirmation of ketosis (BHB >0.5 mmol/L), and standardised symptom questionnaires composed of binary (yes/no) items addressing common side effects such as nausea, constipation or fatigue were employed. Participants were instructed to avoid any additional foods or beverages not explicitly included in the dietary protocol.

In all, 40 patients were deemed sufficient to detect a clinically relevant 15% weight loss (power 0.80, alpha 0.05). Foreseeing a 15% drop‐out, 48 patients were enrolled. Data are expressed as mean and standard deviation or %. Non‐normally distributed variables were log‐transformed. A general linear repeated measures model was used to analyse continuous endpoints. The time × treatment interaction analyses were used to determine groupwise differences. The Pearson χ^2^ test was used for categorical variables. Analyses were performed using SPSS‐27.0 (Armonk, NY, USA). All tests were two‐tailed, and a *p*‐value <0.05 was considered statistically significant.

## 3 RESULTS

Of 62 screened patients, 48 were enrolled; the primary reason for exclusions was difficulty in matching participants across groups. Twenty patients per group completed the study, of which 17 and 15 were female, respectively. Dropouts in the VLCKD group were due to personal reasons (*n* = 3) or adherence difficulties (*n* = 1), while dropouts in the VLCKD+liraglutide group were due to economic constraints (*n* = 2), intolerable constipation (*n* = 1) and personal reasons (*n* = 1). The maximum liraglutide dose reached was 1.8 mg due to limited tolerability or patient preference.

At baseline, groups showed no significant differences in age, gender, weight, BMI, WC, glucose or other parameters, except insulin (*p* = 0.006), HOMA‐IR (*p* = 0.005), and total cholesterol (*p* = 0.047), which were higher in the VLCKD+liraglutide group (Table 1).

**TABLE 1:** Clinical Changes in Patients Following VLCKD and VLCKD with Liraglutide: A Comparative Analysis.

Variable	VLCKD Pre (Mean ± SD or %)	VLCKD Post (Mean ± SD or %)	VLCKD + liraglutide Pre (Mean ± SD or %)	VLCKD + liraglutide Post (Mean ± SD or %)	p1 (baseline)	p2 (over time)
Demographics, anthropometrics, habits						
Age (years)	51.3 ± 8.4	51.3 ± 8.4	49.3 ± 11.8	49.3 ± 11.8	0.54	ns
Gender (*N* Female)	17		15		0.7	n/a
Weight (kg)	98.7 ± 23.4	84.2 ± 19.5	102.2 ± 25.0	81.4 ± 18.3	0.654	**0.013**
BMI (kg/m^2^)	37.1 ± 6.1	31.6 ± 4.9	35.3 ± 7.5	29.0 ± 5.7	0.411	**0.027**
WC (cm)	111.6 ± 15.8	97.0 ± 14.6	116.3 ± 14.9	96.2 ± 11.9	0.349	0.061
Physical Activity (min)	45.0 ± 23.7	67.5 ± 111.8	28.5 ± 12.4	31.5 ± 55.5	0.542	0.19
Biochemistry						
BHB Levels (mmol/L)	0.0 ± 0.0	0.6 ± 0.4	0.0 ± 0.0	1.0 ± 0.3	1	**0.001**
Glucose (mg/dL)	94.9 ± 8.6	92.0 ± 7.8	100.9 ± 10.2	91.6 ± 8.8	0.06	0.121
Insulin (mIU/L)	12.1 ± 7.4	7.4 ± 2.7	17.8 ± 4.2	8.6 ± 1.7	**0.006**	**<0.001**
HOMA‐IR	2.9 ± 1.8	1.7 ± 0.7	4.5 ± 1.2	2.0 ± 0.4	**0.005**	**0.001**
Creatinine (mg/dL)	0.9 ± 0.2	0.8 ± 0.1	0.8 ± 0.2	0.8 ± 0.2	0.243	**0.01**
Sodium (mmol/L)	140.8 ± 1.8	140.7 ± 2.7	140.3 ± 2.2	141.6 ± 2.8	0.401	0.223
Potassium (mmol/L)	4.5 ± 0.4	4.6 ± 0.4	4.3 ± 0.3	4.4 ± 0.3	0.244	0.683
AST (IU/L)	21.4 ± 8.1	18.0 ± 3.5	21.0 ± 4.4	18.8 ± 3.8	0.822	0.621
ALT (IU/L)	28.6 ± 23.5	18.8 ± 5.4	25.1 ± 9.0	18.8 ± 4.5	0.529	0.571
Total Cholesterol (mg/dL)	203.1 ± 29.1	188.3 ± 29.6	222.9 ± 31.8	194.6 ± 32.2	0.047	0.354
LDL Cholesterol (mg/dL)	130.5 ± 36.0	121.1 ± 29.0	141.5 ± 30.5	121.9 ± 31.6	0.305	0.458
HDL Cholesterol (mg/dL)	57.4 ± 17.2	50.5 ± 13.8	56.6 ± 13.4	56.6 ± 10.7	0.876	**0.01**
Triglycerides (mg/dL)	104.5 ± 36.0	84.0 ± 33.2	123.8 ± 44.8	80.3 ± 28.4	0.14	0.101
Urate (mg/dL)	5.2 ± 1.3	5.0 ± 1.3	5.6 ± 1.1	4.8 ± 0.8	0.255	0.145
Symptoms						
Nausea or vomiting (%)	0	0	0	100	1	**<0.001**
Heartburn (%)	10	5	5	5	0.548	1
Constipation (%)	15	35	20	80	0.677	**0.004**
Reflux (%)	15	5	15	25	1	0.077
Muscle weakness (%)	10	15	5	15	0.548	1
Fainting sensation (%)	0	0	0	15	1	0.072
Palpitations (%)	10	10	10	5	1	0.548
Hunger (%)	75	5	100	10	**0.017**	0.548
Mental fatigue (%)	10	15	5	5	0.548	0.292

*Note*: *p*₁, Statistical significance (*p*‐value) for the difference between baseline (pre) values of the VLCKD and VLCKD+liraglutide groups; *p*₂, Statistical significance (*p*‐value) for the difference in changes over time (from pre to post) between the VLCKD and VLCKD+liraglutide groups.

Abbreviations: ALT, alanine aminotransferase; AST, aspartate aminotransferase; BHB, beta hydroxybutyrate levels; BMI, body mass index; HOMA‐IR, homeostasis model assessment of insulin resistance; VLCKD, very low‐carbohydrate diet; WC, waist circumference.

**FIGURE 1:**
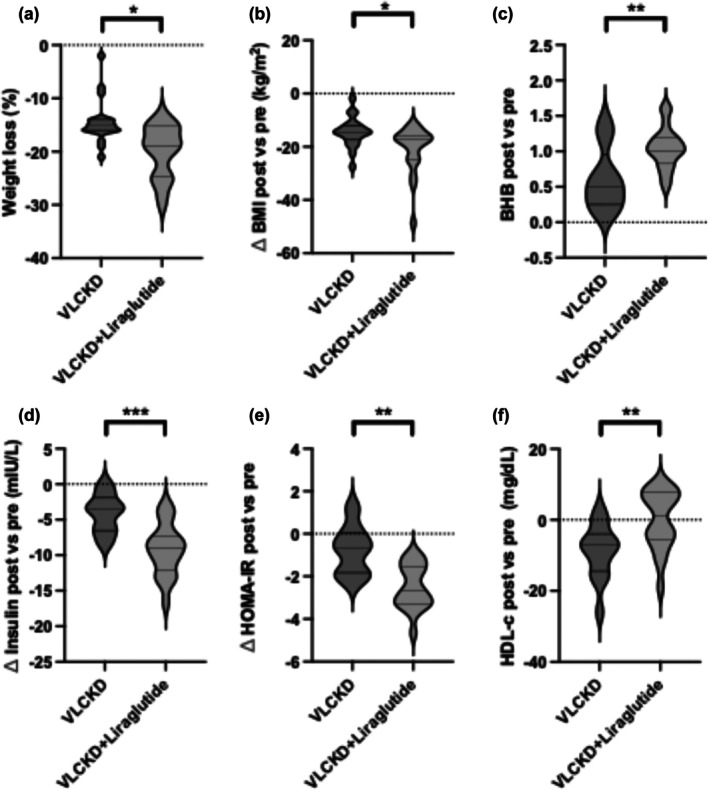
(A–F) Effects of VLCKD and VLCKD plus liraglutide on anthropometric, metabolic and biochemical parameters. Violin plots showing the distribution of change (Δ) from baseline (pre‐treatment) to post‐treatment for the following variables: (A) Body weight (%); (B) Body Mass Index (BMI, kg/m^2^); (C) Beta‐hydroxybutyrate (BHB, mmol/L); (D) Insulin levels (mIU/L); (E) HOMA‐IR index; (F) HDL cholesterol (mg/dL). Each violin represents the full data distribution, with the width corresponding to data density. Medians and interquartile ranges are indicated. Statistical comparisons between groups were performed on the delta values using unpaired two‐tailed t tests. *p* < 0.05 (*), *p* < 0.01 (**), *p* < 0.001 (***)].

Both interventions improved outcomes over time, but VLCKD+liraglutide resulted in a greater reduction in body weight (−20.8 kg vs. −14.5 kg; estimated difference 2.8 kg, 95% CI: −11.1 to 16.7; *p* = 0.013 for time × group interaction) (Figure 1A), and in BMI (−7.3 vs. −5.5 kg/m^2^; estimated difference 3.26 kg/m^2^, 95% CI: −0.59 to 7.1; *p* = 0.027 for time × group interaction) (Figure 1B). Interestingly, 95% of the VLCKD+liraglutide group reached a weight loss of 15% and over, whereas only 65% of the VLCKD group reached the threshold (*p* = 0.048). WC reduction was comparable, though slightly greater in the combination group. Beta‐hydroxybutyrate (BHB) levels increased in both groups but were higher in the VLCKD+liraglutide group (1 ± 0.3 vs. 0.6 ± 0.4 mmol/L; estimated difference −0.44 mmol/L, 95% CI: −0.619 to −0.261; *p* = 0.001 for time × group interaction) (Figure 1C). Sustained ketosis (BHB >0.5 mmol/L) was achieved by all patients in VLCKD+liraglutide vs. 80% in VLCKD alone (*p* = 0.035). Hunger decreased in both groups similarly (Table 1). Improvements in insulin and HOMA‐IR were greater with liraglutide (insulin: estimated difference 2.5 μU/mL, 95% CI: −3.7 to 8.6; HOMA‐IR: 0.52, 95% CI: −0.82 to 1.86; both *p* ≤ 0.001 for time × group interaction) (Figure 1D,E), while glucose reductions did not significantly differ between groups (Table 1). No symptomatic hypoglycaemia was reported. Noteworthy, no patients with diabetes were enrolled.

Regarding safety, liver and renal parameters remained within safe ranges. Creatinine slightly decreased in the VLCKD group compared with VLCKD+Lira (estimated difference −0.05 mg/dL, 95% CI: −0.16 to 0.06; *p* = 0.01 for time × group interaction), whereas aminotransferases were unchanged. Total and LDL cholesterol improved similarly in both groups, whereas HDL cholesterol remained stable only in the VLCKD+liraglutide group (estimated difference 7.65 mg/dL, 95% CI: −1.5 to 16.8; *p* = 0.01 for time × group interaction) (Figure 1F). Triglycerides, uric acid, and electrolyte levels showed no significant differences (Table 1). Nausea/vomiting (100% vs. 0%, *p* < 0.001) and constipation (80% vs. 35%, *p* = 0.004) were more frequent with liraglutide (Table 1), while other adverse events occurred similarly across groups. No specific patterns in tolerability were observed, and no clear characteristics could be identified to distinguish participants with lower tolerability from those who benefited most from each treatment.

## 4 CONCLUSIONS

This study is the first to evaluate the effects of combining a GLP‐1 RA with a VLCKD. Both interventions led to weight loss and metabolic improvements; however, adding liraglutide enhanced these outcomes. The greater weight reduction observed with liraglutide is likely due to its appetite‐suppressing effects, which complemented the VLCKD by potentially improving dietary adherence, as indicated by higher BHB even compared with similar studies.^14^ The weight loss achieved with the combination therapy was markedly greater than what is typically observed with liraglutide 3 mg alone.^10^ Additionally, insulin sensitivity improved more markedly in the combined group, consistent with liraglutide's effects on insulin secretion and glucagon suppression.^15^

The combination therapy was also associated with increased gastrointestinal effects, and no participant followed the standard weekly dose uptitration. For some, the lower dose was sufficient; for others, side effects limited dose escalation, raising concerns about the combination tolerability. Interestingly, the combination led all participants to develop nausea or vomiting at some point, likely because both interventions can independently lead to these symptoms, possibly due to delayed gastric emptying.^6^, ^16^, ^17^ It is therefore plausible that a VLCKD may amplify the GI adverse events commonly occurring during GLP‐1 RA treatment, although this should be further investigated in the future. Despite this, biochemical safety outcomes remained stable, supporting the safety of combining liraglutide with a VLCKD, at least for short‐term use, and the dropout rate between the two groups was identical, confirming that the adverse events did not compromise adherence or overall treatment outcomes.

The study had some limitations, including a relatively small sample size, although a priori calculation was performed and met. The absence of long‐term follow‐up data limits conclusions about sustained effects, although this duration is relatively extended for ketogenic interventions. The use of GLP‐1 receptor agonists following carbohydrate reintroduction, as a strategy to sustain weight loss and preserve metabolic health benefits, has not been investigated and warrants evaluation in future studies. This was a non‐randomised, pragmatic study design allowing patients to choose their preferred intervention, potentially introducing selection bias. However, permitting patient choice likely improved real‐world adherence.^18^ Real‐world data are indeed essential to understanding how interventions perform outside of tightly controlled experimental conditions, particularly in outpatient clinical practice where patient adherence, resource constraints and treatment variability often differ significantly from RCT environments. In addition, the lack of a liraglutide‐only control group limits the interpretation of liraglutide's independent effects. Moreover, no body composition analysis was done, preventing differential muscle versus fat loss assessment. Concerning hypoglycaemia, patients only reported symptoms, and no episodes were identified; however, the absence of continuous glucose monitoring means that asymptomatic episodes could have been missed. Lastly, since no patient reached the maximum liraglutide dose approved for obesity treatment, conclusions regarding the efficacy and safety of higher doses cannot be drawn.

The study also had important strengths. As the first investigation of a combined GLP‐1 RA and VLCKD approach, it provides valuable preliminary evidence. Demographic and clinical characteristics matching allowed accurate comparisons. Ketosis monitoring and access to dietitian support improved dietary adherence and data quality. Moreover, sustaining ketosis over 4 months, longer than most studies, provided unique insights into the long‐term direct effects of nutritional ketosis.

In conclusion, combining liraglutide and VLCKD promotes enhanced weight loss and metabolic improvements, albeit with a higher rate of adverse events, warranting careful clinical monitoring. These findings are particularly timely, as liraglutide is approaching patent expiration and will likely become significantly more affordable, potentially with price reductions of at least 50%, as observed for similar agents in the past.^19^ Meanwhile, VLCKDs are increasingly underutilised in favour of newer pharmacological options. If Tirzepatide 15 mg induces a 20% weight loss over 12 months^20^ with out‐of‐pocket cost in Italy of ~€6500 for this time length,^21^ achieving a similar 20% weight loss in a much shorter time with a regimen combining VLCKD^22^ and liraglutide as per our study would cost ~€2300. Despite both liraglutide and VLCKD approaches being at risk of underuse in the current therapeutic landscape, our results suggest that their combination represents a highly cost‐effective and clinically valuable strategy for obesity management.

## PEER REVIEW

The peer review history for this article is available at https://www.webofscience.com/api/gateway/wos/peer-review/10.1111/dom.16658.

## Data Availability

Data will be made available upon reasonable request to the corresponding authors.
